# Multi-criteria decision making-based waste management: A bibliometric analysis

**DOI:** 10.1016/j.heliyon.2023.e21261

**Published:** 2023-10-24

**Authors:** Shahab Saquib Sohail, Ziya Javed, Mohammad Nadeem, Faisal Anwer, Faiza Farhat, Amir Hussain, Yassine Himeur, Dag Øivind Madsen

**Affiliations:** a Department of Computer Science and Engineering, School of Engineering Sciences and Technology, Jamia Hamdard, New Delhi 110062, India; b Department of Computer Science, Aligarh Muslim University, Aligarh 202002, India; c Department of Zoology, Aligarh Muslim University, Aligarh, 202002, India; d Edinburgh Napier University, United Kingdom; e College of Engineering and Information Technology, University of Dubai, Dubai, United Arab Emirates; f School of Business, University of South-Eastern Norway, 3511 Hønefoss, Norway

**Keywords:** Waste management, Multi-Criteria-Decision-Making (MCDM), Bibliometric analysis, Circular economy, Sustainability

## Abstract

Waste management is a complex research domain. While the domain is challenging in terms of content, it is also a diverse and cross-disciplinary research subject. One of its important components includes efficient decision-making at various levels and stages. Therefore, Multi-criteria decision-making (MCDM) techniques have found decent applications in this domain. The field of MCDM techniques-based waste management has been examined using bibliometric analysis in this paper in order to report a systematic overview of the trends and advancements in this area of study. The Scopus database provided 216 publications on the aforementioned subject written between 1992 and 2022. The 216 articles include 56 countries, 158 institutions, and 160 authors. Furthermore, Asian countries, including India, Iran, and China, dominate this field of study. The geographical disparity in the output of publications is visible. Journal of cleaner production, Waste Management and Waste Management and Research are the major journals publishing on MCDM techniques-based waste management research. Given that majority of the articles include multiple authors, it can be said that there is a lot of collaborative research in this area. Overall, the current study provides a thorough analysis of the development in the domain of waste management using MCDM techniques. The trend suggests that it will continue to be a focus of research for academicians, environmentalists and policymakers in the future.

## 1 Introduction

Sustainability and waste management are fundamental to ensuring a harmonious future for our planet. As global populations rise and urbanization intensifies, the sheer volume of waste we produce presents significant challenges to our ecosystems and natural resources [1]. Proper waste management is not just about disposing of materials but also about reducing consumption, reusing, and recycling, all of which contribute to a circular economy. Emphasizing sustainability means we acknowledge the finite nature of our resources and strive to minimize our environmental footprint [2]. By prioritizing these principles, we safeguard our environment, ensure resource availability for future generations, and cultivate a healthier, more equitable world [3].

The ever-increasing volume of waste produced has been one of the primary concerns for any society as it also causes environmental contamination. The increase in urban population and industrial and economic growth are the main factors behind it [4]. As a result, achieving the development goals of a state and creating a more sustainable society depends heavily on effective waste management procedures [5], [6].

If we focus only on Municipal Solid Waste (MSW), 2.9 billion urban residents produced 0.64 kg of MSW per person per day (0.68 billion tonnes per year) in the early 2000s. In 2017, 3 billion people produced 1.2 kg per person per day (1.3 billion tonnes per year) [7]. By 2025, it is anticipated that 4.3 billion people will live in cities, producing 2.2 billion tonnes of municipal solid trash daily (or 1.42 kg per person) [8]. Poor waste management can seriously harm the environment and people's health, especially when it is poorly placed, i.e., close to densely inhabited regions, water supplies, and sewage systems. It increases greenhouse gas (GHG) emissions, worsening the effects of global warming. Moreover, the expenses associated with improper waste management are greater than the initial investment in suitable storage and disposal facilities [7]. Improperly handled waste has significant negative consequences on health, the local and global environment, and the economy. Effective waste management is greatly influenced by the categories of waste that the population produces. Socioeconomic status, the size of the household, and even the seasons have an impact on waste composition [9]. It is challenging to manage the treatment and disposal of garbage in a way that is both economically and environmentally viable since waste composition and quantity are dynamic. It is necessary to start with a precise definition of the challenge in order to successfully implement a decision-making process that results in functional and sustainable waste disposal methods [10]. A thorough examination of the population needs planned development directions, and implementing strategies is necessary for choosing the most efficient scenario and providing recommendations [11]. The health and well-being of the people would be impacted differently by various scenarios, which would also differ in terms of cost and usually had aims that were in conflict [12]. Furthermore, a scenario that works well to manage the waste at one location may be inappropriate or ineffective for another location, depending on the makeup of the waste produced [13], [14]. Authorities need quick and efficient tools to model the most relevant choice alternatives that satisfy the pre-selected conditions for the area under consideration and then choose the best among them [15], [16].

Multi-Criteria Decision Making (MCDM) is a set of methodologies to provide solutions for complex problem decision-making processes. It offers solutions to issues involving a wide range of indicators and takes into account all relevant criteria linked to waste management issues [17]. These issues involve a number of management options that ought to be assessed simultaneously in light of several different choice criteria [18]. MCDM approaches have several advantages over other conventional decision-making tools. Many researchers have outlined the benefits and limitations of MCDM approaches [19]. In a nutshell, the main strength of MCDM procedures is their adaptability to the judgment of the decision-making team, which investigates the best choice by distributing performance weights and scores. They have the ability to compare alternatives to a variety of criteria that are measured using various (both quantitative and qualitative) modes [20]. Additionally, they can be used to determine the most preferred alternative, shortlist alternatives for further in-depth examination, rank many alternatives, or separate acceptable from undesirable possibilities. The performance matrix, where each row represents an option and each column describes the performance of the option against each criterion, is another crucial component of MCDM. The performance matrix can be used to compare the outcomes of different pair-wise candidates for each of the selected criteria before making a final decision [15]. There are a number of MCDM methods available in the literature. The analytical hierarchy process (AHP), ELECTRE, TOPSIS, PROMETHEE, and their variations are often employed in waste management challenges [9].

Waste management systems are complicated and require many decision-making processes at various levels. MCDM approaches can be used for various decision-making processes, including the selection of waste treatment, disposal, and recycling technologies, landfill location selection, Waste plant location selection, and waste treatment scenario selection [21]. Because MCDM techniques are simple to apply as group decision-making tools, a wide variety of experts (from various backgrounds) can be included in the decision-making process. Another benefit of MCDM is that it can handle ambiguity and uncertainty in expert opinions and real-world parameter variations [22], making it ideal for decision-making.

Though there are a few reviews are there, we are not aware of any reviews that illustrate the overall evolution of the state-of-the-art in recent decades or that describe the present publication landscape of waste management research using MCDM approaches, despite the availability of scientific publications and reviews. To fill this knowledge gap, we conduct a bibliometric analysis of articles detailing MCDM techniques for waste management published between 1992 and 2022. Research trends, emerging topics, and novel dynamics across research areas, nations, and researchers can be discovered with the aid of bibliometric analysis [23]. The primary research questions we are aimed at answering includes – i) trend analysis of publications, ii) citation and co-citation analysis, iii) the top avenues (journals/conferences), countries/regions, and institutions, iv) top publishing and highly cited authors and their scientific collaborations, and v) most emerging keywords/research topics currently in considerations and prospective for future.

This article delves into Multi-Criteria Decision Making (MCDM) in waste management, encompassing a thorough analysis of influential articles, collaborative networks among authors, institutional partnerships, and emerging research trends. Notably, articles with over 100 citations highlight techniques like TOPSIS, ELECTRE, and PROMETHEE I/II, while co-citation analysis reveals authors with close research ties, including prolific collaborations by Kahraman and Karagiannidis. Institutional collaboration networks showcase the most active partnerships, with the Ferdowsi University of Mashhad's Department of Civil Engineering leading. Keywords and co-occurrence patterns underscore prevailing research themes, with terms like “circular economy” and “pyrolysis” signifying future directions. The study's comprehensive timeline of keyword occurrence underscores evolving research trends, spotlighting emerging areas like sustainable practices and waste disposal methods. The present analysis provides a more comprehensive picture of the research landscape and, in addition to educating academics, it can motivate governments, financial organizations, and funding organizations to support innovative approaches to address unanswered research questions and assist in the filling of relevant research gaps. The organization of the paper is as follows. Section 2 discussed the review/survey works in the field of MCDM-based waste management. Section 3 describes the Research Methodology of the current work, including the data extraction approach and visualization tool used. The overall bibliometric analysis of various indicators is presented in Section 4. Section 5 presents the concluding remarks.

## 2 Background and related work

### 2.1 Multicriteria decision making (MCDM)

There are some significant numbers of reviews on waste management using MCDM approaches. Table 1 presents a summary of each review work on key points such as review article, no of articles reviewed, type of review, key findings, key recommendations, and waste domain. Very few review articles followed a systematic methodology and hence may miss several relevant works in this direction. The main focus of this study is to perform a bibliometric study on MCDM approaches applied to waste management. The bibliometric study is used for several reasons, such as to look for the intellectual structure of a domain, to explore emerging trends in the field, collaboration patterns, and others.

**Table 1:** Summary of the review works on waste management.

Review Article	# Reviewed articles	Review type	Key Findings	**Key Recommendations**	**Waste domain**
Santos et al. [15]	20	Semi-systematic	**Table:** The criteria are weighted mainly through experts, then decision-makers and on a few occasions by affected population	**Table:** The study recommends several key points that can be served as a checklist when applying MCDA to address waste management related to marine and terrestrial plastic.	Plastic waste

Guillermo Garcia-Garcia [16]	Not mentioned	Unsystematic	**Table:** Decision-makers generally set the evaluation criteria as very subjective	**Table:** To optimize solid waste management, various methods and tools should be integrated, or customized methods need to be developed	**Table:** Solid waste management

Torkayesh et al. [17]	113	Semi-systematic	**Table:** Very few studies are presented to integrate LCA and MCDM in assessing the sustainability of waste management systems	**Table:** For comprehensive sustainable waste management, goal should be defined first, followed by scope definition and other steps.	**Table:** Waste management

Abu et al. [18]	13	Unsystematic	**Table:** Stakeholders as decision-makers should be creatively engaged for the lasting success of FW management	**Table:** a model that combines Life Cycle Assessment (LCA), life cycle costing (LCC), and MCDM needs to be introduced.	Food waste

Marinello and Gamberini [19]	44	Systematic	**Table:** Including environmental criteria in decision-making processes enhances the relevance of theWEEE management.	**Table:** Developments should be made to transfer and capitalization of the existing methods.	**Table:** Electrical and Electronic Equipment waste management

Coelho et al. [20]	260	Systematic	**Table:** MCDM in solid waste management mainly addresses the problems of municipal solid waste concerning facility location or management strategy	**Table:** GIS should be used in association with MCDM for the problems specific to spatial data.	**Table:** Solid waste management

Soltaniet al. [21]	68	Semi-systematic	**Table:** AHP is the most common MCDM technique focusing on multiple stakeholders	**Table:** different types of compromises should be considered while focusing problems on multiple stakeholder decision-making.	**Table:** Multiple solid waste management

Achillas et al. [22]	44	Unsystematic	**Table:** The work provides a list of practical applications of MCDA techniques which are used in solving real-life problems of waste management .	**Table:** The developed models should be validated, and their uniqueness needs to be assured.	**Table:** Waste management

MCDM has been applied to several waste streams, such as plastic waste, municipal solid waste, wastewater, electrical and electronic equipment, construction and demolition waste, nuclear/radioactive waste, food waste, and others. Eight review studies have been considered in this section. Three are related to solid waste management, one is concerned with plastic waste management, one is related to food waste, one focuses on electrical and electronic equipment waste, and two are general waste management reviews.

Santos, Dias, Cunha, and Marques [24] present an overview of existing MCDM approaches for managing marine and terrestrial plastic waste. The study depicts a comprehensive list of alternatives and criteria applied in different studies, which can be served as a checklist when using MCDM for marine and terrestrial plastic waste management. In another study [25], a review of MCDM methods and tools is discussed to assess solid waste management systems, where it is found that the Analytic Hierarchy Process (AHP) followed by Multi-Attribute Utility Theory (MAUT) and Outranking procedures and the Technique for Order of Preference by Similarity to Ideal Solution (TOPSIS) are most popular methods.

In another study Torkayesh et al. [9] review MCDM and life cycle assessment (LCA) approaches that evaluate sustainability. The authors reviewed 113 papers in which a significant number of studies applied AHP to assign weight to the decision criteria and assess options for alternative waste management. They further highlighted that 47% of studies applied AHP, 13% TOPSIS, VIekriterijumsko KOmpromisno Rangiranje (VIKOR) method is applied by 7% of studies while 5% studies applied DEMATEL followed by some portions of studies applied by ELECTRE, BWM, Analytic Network Process (ANP) and others.

A review work [26] primarily focuses on Food waste (FW) management and analyses MCDM techniques in modeling and investigating decision-making, such as the acquisition of suitable waste treatment sites or methods used in the circumstances with multiple stakeholders. While the focus of the review [27] is to analyze the most popular MCDM methods applied to the management of waste electrical and electronic equipment (WEEE) and determine how they are improving the management strategies in the entire supply chain. For this purpose, 44 papers are identified through a methodological protocol.

A comprehensive literature review [16] on the application of MCDM in solid waste management was presented that ultimately offered a critical analysis of the current practices. In another review [28] in the same domain of solid waste management, the authors presented research on the application of MCDA in solving multiple solid waste management problems with more emphasis on the works that have included multiple stakeholders. The authors further classified the studies depending on the involvement of groups of stakeholders. A separate study [15] provides a list of practical applications of Multi-Criteria Decision Analysis (MCDA) techniques, which are used in solving real-life waste management problems, and the criteria used in those applications depend on the nature of the problems. The review also highlights the advantages and disadvantages of applying MCDA techniques in waste management problems compared to other available options.

### 2.2 Bibliometric analysis

**Figure 1:**
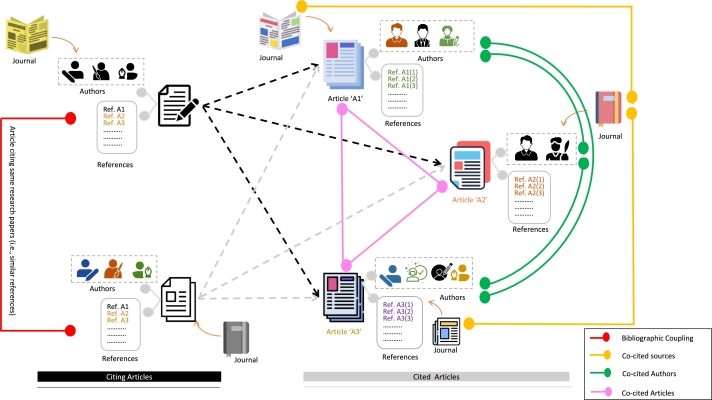
An illustration of the components of a Bibliometric study.

Bibliometric review has been widely used in the literature across disciplines of studies. A Scopus search with queries “bibliometric studies” or “bibliometric analysis” or “bibliometric review” on Aug 20, 2023, returns more than 21,977 articles. More interestingly, approximately 10000 articles on bibliometrics have been published in 2022 and 2023. However, a generic illustration that helps understand key terms and components of this study is vital and shall be in the interest of researchers and readers. To this end, Fig. 1 illustrates the different aspects of bibliometric analysis. In general, an article is published in a journal, where each article, in addition to the contents, has author(s) and references. References are articles that are cited and referred to in an article. If the references of two articles are mostly common, i.e., these articles have a similar tendency of citing articles, these two are called bibliographic coupling, as shown in the red line in the figure.

In the Fig. 1, citing articles and cited articles are shown separately; if two or more articles are cited by the same articles, these two are called co-cited references (or articles). This has shown in pink color. Similarly, if authors of two articles are cited by the same articles, these authors are termed as co-cited authors, as shown in green color. In the same way, if two journals in which articles are published are cited by the same journal, are referred to as co-cited sources. These are some of the primary units used for bibliometric analysis. The further explanations are provided in the respective sections.

## 3 Research methodology

### 3.1 Data extraction

The recent growth in the bibliometric study has attracted researchers to explore further in the concerned area of research. These kinds of research are helpful, particularly in identifying suitable avenues to publish, potential researchers working in an area for possible collaboration, and future keywords promising to explore further. We have followed the bibliometric analysis approach as suggested in a pioneer work [29], [30] and more recently in [31].

We searched different combinations of the keywords “waste management” AND “MCDM” OR “MCDM” on November 2022 in the SCOPUS online database to identify publications. As a result of the searches made, we have obtained 297 results. Further, we processed the available responses for duplicity and relevancy. To this end, we have excluded the articles for the ‘review’, ‘conference review’ and ‘notes’ categories. Moreover, manual screening is aimed at enhancing the quality of the manuscripts to be considered through reading the abstract and investigating the content to understand the theme of the articles to exclude irrelevant articles.

Hence, keeping the relevancy as a prime factor for consideration of the articles. In addition to the exclusion criteria as discussed, we have also not considered articles in any language other than English. Finally, we have included 216 results for experimentation. For the bibliometric review analysis, we have kept publications mentioning the word “waste management” AND “MCDM” OR “MCDM” in the title, abstract, or keywords. The search results were limited to journal articles as well as conference proceedings and book chapters but must have been written in English and the publication date is on or before November 2022.

Initially, the following components of the retrieved articles were used for the assessment. a) Organization b) Countries/Regions c) Number of publications per year. d) Avenue: Journals/Conference proceeding e) Total Citations f) Keywords Once this information is gathered and stored, author names with their affiliations, i.e., affiliated institutions and countries, are extracted.

### 3.2 Data visualization tool (VOSviewer)

For bibliometric studies, we have exploited VOSviewer [32], [33]. It is a software application that allows one to create maps based on (research) network data and then enable visualizing and exploring maps. VOSviewer has been extensively used for bibliometric analysis in various domains, including business [34], [35], [36], computer science [37], [38], [39], agriculture [40], [41], [42], social science [43], [44], [45], [46] and medicine [47], [48], [49]. These analyses were conducted using the default parameters. The font size of the words on the map represents the frequency with which the terms appear (Multiple appearances in the same publication are counted as one). If two words co-occurred more frequently in the evaluated articles, they are more closely related. The VOS clustering method was applied to cluster topics into different groups, where each cluster is marked with a different color [50], [51]. The interpretation of the visualizations can be briefly summarized as follows: The frequency of occurrence is represented by the size of the circles and the font of the label represents, the colors represent clusters, and relatedness and similarity are indicated by the distance between two circles [52], [53]. The x-axis and y-axis have no special meaning; the maps may be freely rotated and flipped [54], [55], [56].

### 3.3 Bibliometric indicators

While performing bibliometric analysis, many indicators have been used in the literature. Total article, Total citation, Total link strength, average citations per article (ACPA) and Hirsch index (H-index) are a few metrics frequently being used for bibliometric studies. For instance, H-index is widely recognized as a measure of the quality and quantity of research avenues and authors. Furthermore, ACPA is a well-accepted measure for identifying the research impact of a particular work, author or publication avenue, etc. We have considered all these indicators in this bibliometric study. Moreover, citation and co-citation are well-regarded for exploring the scientific impact and theme of the study under consideration. In addition to this, co-authorship and co-occurrence have also been explored to investigate scientific collaboration.

## 4 Findings and critical discussions

The total duration for which articles have been taken into consideration, including the sources from which the articles are sourced, the total number of authors contributing to the topic, and relevant meta-information, all of which are summarized in Table 2. In the subsequent subsections, we provide explicit visual representations through diagrams and tables, showcasing the current publication trends and discussing insightful aspects of various topics under consideration.

**Table 2:** Meta information about bibliometric analysis on “MCDM based waste management”.

MAIN INFORMATION ABOUT DATA	
Timespan	1992-2022
Sources (Journals, Books, etc.)	125
Documents	218
Annual Growth Rate %	13.18
Document Average Age	5.44
Average citations per doc	48.76
References	10092
DOCUMENT CONTENTS	
Keywords Plus (ID)	1582
Author's Keywords (DE)	596
AUTHORS	
Authors	677
Authors of single-authored docs	6
AUTHORS COLLABORATION	
Single-authored docs	6
Co-Authors per Doc	3.59
International co-authorships %	24.77
DOCUMENT TYPES	
article	187
book chapter	6
conference paper	23

### 4.1 Publication trend analysis (PTA)

Publication trend analysis (PTA) is aimed at analyzing patterns and changes in the volume and type of publications produced over time. This type of analysis can provide insights into the growth and development of a research field, as well as trends in research productivity, impact, and collaboration. It helps identify research hotspots and areas of growth, assess research productivity and impact, compare the performance of institutions, researchers, and countries, forecast future trends and identify emerging fields, etc.

**Figure 2:**
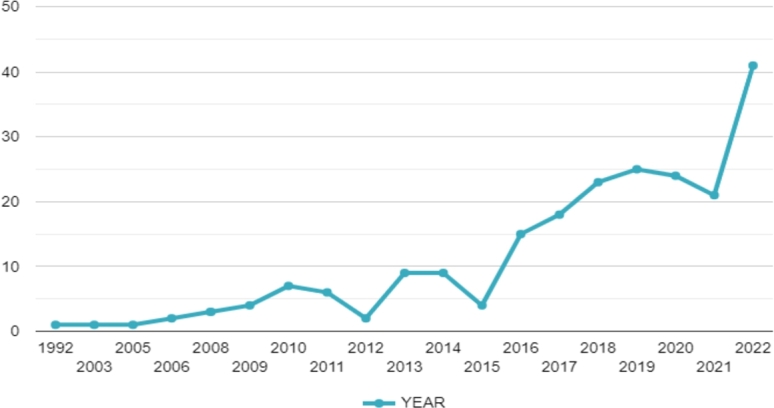
Publications Trend analysis indicates an early increment followed by a zigzag curve which recently has seen a sharp increase in publications through time.

Fig. 2 displays the trends of articles published between 1992 and 2022, including those related to MCDM-based waste management. While MCDM-based waste management articles were first published in 1992, none were published between 1993 and 2002. The number of annual publications experienced sluggish growth from 2003 to 2009, averaging two articles per year. However, by 2015, the number had increased to four articles per year, and a significant turning point was reached in 2016 when over 15 publications were released annually. Although there was no continuous distribution of publications thereafter, the data showed a noteworthy increase, particularly from 2016, with more than 77.31% of papers published after that year. Between 2016 and 2021, the number of articles published annually increased to 21. Notably, the number of publications in 2022 doubled, with an average of 41 articles per year, indicating the growing influence of this field. The recent growth pattern suggests an increasing interest in waste management research by academia, and this attraction is expected further to boost research in this area in the near future.

### 4.2 Top avenues (journals/conferences), authors, institutions and countries/regions

#### 4.2.1 Journal analysis

In accordance with their article counts, Table 3 presents information on the top 10 journals. The Journal of Cleaner Production is the leading journal in terms of total citation, H-Index, and article count. Waste Management comes in second in terms of publications, with “Waste Management and Research” ranking third. Environmental Science and Pollution Research Resources have published nine articles on the relevant topic, whereas “Resources Conservation and Recycling” and “Sustainability” have each published eight. The journal with the most promising high citation per article ratio is “Waste Management,” with a score of 69.86667. According to the H-Index, the top journal is the “Journal of Cleaner Management.

**Table 3:** Top journals ordered according to the article count.

Journal	Article Count (AC)	Total Citation (TC)	ACPA	Coverage	H-Index
Journal of Cleaner Production	16	996	62.25	1993-2021	232
Waste Management	15	1048	69.86667	1983-2021	182
Waste Management and Research	13	252	19.38462	1983-2021	86
Environmental Science and Pollution Research	9	58	6.444444	1994-2021	132
Resources Conservation and Recycling	8	383	47.875	1988-2022	150
Sustainability	8	109	13.625	2009-2021	109
Journal of Environmental Management	4	271	67.75	**Table:** 1973, 1975, 1977-2022	196
Journal of Intelligent and Fuzzy Systems	4	4	1	1993-2021	64
Environmental Earth Sciences	3	154	51.33333	2009-2021	130
Environmental Monitoring and Assessment	3	19	6.333333	1981-2021	122

The search query produces 114 different journals with 216 articles. Almost 21% of total articles were published by three journals viz., Journal of Cleaner Production, Waste Management and Waste Management and Research. Approximately 55% of articles were published in 24 journals as a whole. 89 different journals have published just one article each. It can be concluded that very few journals have published articles related to undergoing surveys, which shows either the scope of the journal does not fully include the surveyed domain or researchers are not very active in this field. In addition to the journals, as mentioned above, there are conferences where a few articles on waste management have been published. Materials Today Proceedings, has 2 articles published in it with H-index 56, whereas ACM International Conference Proceeding Series, with H-index 128, has published only one article. AIP conference proceeding, E3s Web Of Conferences, International Multidisciplinary Scientific Geo-conference Surveying Geology And Mining Ecology Management SGEM, and IOP Conference Series Earth And Environmental Science are the other avenues having published one article each with H-index 75, 28, 24 and 34, respectively.

#### 4.2.2 Country analysis

**Figure 3:**
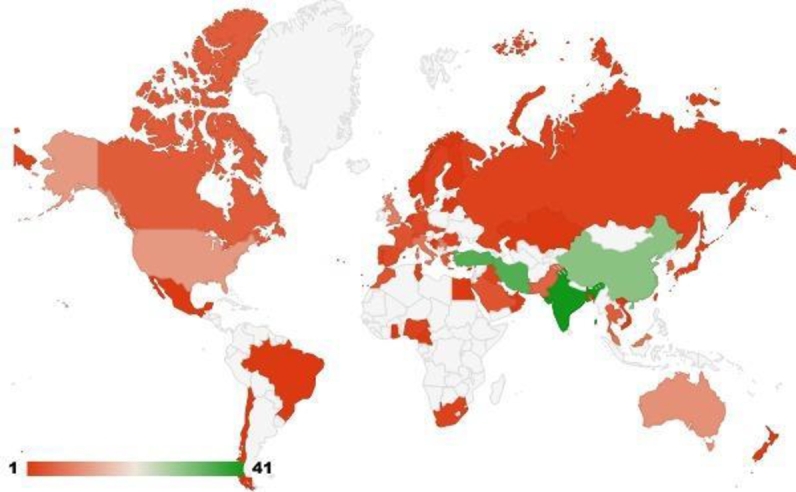
Publications distribution among countries Analysis of publication frequency via number of articles and contributing countries.

Articles on waste management using MCDM techniques come from 56 different territories/nations. The top ten countries/territories, along with total citations, are shown in Table 4. Four (04) of the top 10 countries are Asian. It could be because, as Asian countries are aiming at industrial and economic growth, efficient waste management has become essential. Therefore, these countries could be developing strategies for their specific situations. The distribution of the contributing territories has also been shown in Fig. 3. India, with 41 publications, has the most articles overall, as seen above. Next, with 35 publications, are Iran and Turkey. Then, with a total of 30 publications, China came in third.

**Table 4:** Top 10 countries, sorted in order of their articles count.

Country	Article Count (AC)	Total Citation (TC)	ACPA
India	41	796	19.41
Iran	35	565	16.14
Turkey	35	959	27.4
China	30	1019	33.96
United States	15	559	37.26
Australia	11	332	30.18
Serbia	9	102	11.33
United Kingdom	9	296	32.88
Italy	8	188	23.5
Malaysia	8	243	30.37

India tops the list with 41 publications, but China has taken the top spot according to maximum citations. With 1019 citations from 30 articles, the highest value of average citations per article is 37.26 for USA, followed by China with a value of 33.96. UK (32.88) is in the third place, followed by Malaysia (30.37) and Australia (30.18) respectively.

#### 4.2.3 Institution analysis

A total of 160 institutes produced research on waste management using MCDM approaches. Out of them, 55.7% of institutes (88/158) contributed only one (01) article each, and 26.6% (42/158) generated two (02) articles each. Table 5 displays an examination of publications by affiliation of the top 10 institutes. Galatasaray Üniversitesi, Turkey, and Islamic Azad University, Iran have the maximum number of publications (total 9). University of Tehran, Iran came second with seven (07) publications. With a total of six articles, Yildiz Technical University, Turkey is in third position, followed by Tongji University, China with five (05) articles. The institutions with the highest productivity are looked at in this section. Tongji University of China, which had the most citations with five documents, was placed in first place, according to the indicator of total citations. With 290 citations Shanghai University, China comes in second followed by Turkey's Yildiz Technical University (276 citations). Ferdowsi University of Mashhad, Iran has the least number of citations in this list (72 citations). It is noteworthy that while processing the data, the authors found 3 affiliations associated with Islamic Azad University. Seven (07) affiliations were Islamic Azad University, four (04) were Islamic Azad University, North Tehran Branch, and five (05) were Islamic Azad University, Science and Research Branch; making a total of 16 affiliations. However, after manual screening, we found that these 16 affiliations were mentioned in 9 unique publications i.e. some publications had more than one affiliation of Islamic Azad University. Therefore, we have considered nine (9) publications related to Islamic Azad University.

**Table 5:** Top 10 institutes, sorted in order of their articles count.

Affiliation	# of published articles	Citations
Galatasaray Üniversitesi, Turkey	9	232
Islamic Azad University, Iran	9	256
University of Tehran, Iran	7	230
Yildiz Technical University, Turkey	6	276
Tongji University, China	5	548
Ferdowsi University of Mashhad, Iran	4	72
Bahçeşehir Üniversitesi, Turkey	4	128
Shanghai University, China	4	290
Shanghai Maritime University, China	4	115
University of Belgrade, Serbia	4	71

It is important to note that out of five, three institutes are from Turkey, making Turkey a key center of this research domain. It is also evident from Table 4 and Table 5. However, Chinese institutes seemed to produce more productive research as the number of citations is more (refer Table 4 and Table 5. Though India tops the list of maximum number of articles produced (4), none of its institutes makes it into Table 5, suggesting that the waste management research using MCDM techniques is distributed through various Indian Institutes. From an average citation per article perspective, Tongji University, China has a maximum value of 109.6 (548/5).

It would be useful to categorize all institutes which have applied MCDM techniques in waste management research into academic (such as universities), private firms (such as industry), or governmental bodies (policymakers). Such a distinction could be used to determine whether the research is firmly rooted in the academic community only or includes stakeholders and policymakers also. However, since related data is not available in the database and must be checked manually, this division will take a lot of time.

#### 4.2.4 Author analysis

Table 6 presents the list of the top ten authors along with number of publications in the domain of waste management using MCDM methods, total citations (TC), and H-index. Chauhan, A., Dursun, M., Karadayi, M.A. and Liu, H.C. have published five articles and You, J.X. has published four. It is also noteworthy that Dursun & Karadayi and Liu & You are co-authors in their papers. Out of them, Liu is the most prominent scholar in the field with 389 citations and 45 H-index values, followed by You with 276 citations and 29 H-index. Of all the authors, Liu, H.C., has received the most citations. Dursun & Karadayi has the least number of citations.

**Table 6:** Top 10 authors, sorted in order of their articles count.

Rank	Author	# of published articles	H Index	Total Citations (TC)	ACPA	Country
1	Chauhan, A.	5	11	216	43.2	India
2	Dursun, M.	5	10	157	31.4	Turkey
3	Karadayi, M.A.	5	7	157	31.4	Turkey
4	Liu, H.C.	5	45	389	77.8	China
5	You, J.X.	4	29	276	69	China
6	Ali, Y.	3	25	15	5	Pakistan
7	Asefi, H.	3	10	92	30.66	Australia
8	Ertugrul, K.E.	3	29	06	2	Turkey
9	Joshi, S.	3	23	209	66.33	India
10	Jozi, S.A.	3	14	30	10	Iran

### 4.3 Co-citation analysis and bibliographic coupling

Citation and co-citation analysis in the bibliometric study are methods for measuring the impact and interrelationships between publications. Citation analysis involves tracking the number and quality of citations a publication receives from other academic works. This provides a measure of the impact and influence of a particular publication or researcher. In contrary to this, co-citation analysis involves examining the co-occurrence of citations between two or more publications. This can be used to identify related research areas, emerging fields, and the relationships between researchers and institutions. Together, citation and co-citation analysis provide valuable insights into the academic landscape, including the spread of ideas and the relationships between researchers, institutions, and disciplines. Furthermore, Co-citation analysis also helps in determining the most important articles in a study subject, as shown in Fig. 5, co-citations of the scientific documents. A co-citation network is formed when two publications appear together in the reference list of another publication. The co-citation analysis excludes current or specialized articles from its subject clusters and only considers sources that have been highly cited. The benefit of using co-citation analysis is that, in addition to finding the most influential publications, one can also discover thematic clusters. Here, the thematic clusters are derived based on the cited publications. However, co-citation analysis concentrates only on highly-cited publications and leaves publications that are recent or niche out of their thematic clusters. In that sense, co-citation analysis is suitable for researchers working in the area of waste management using MCDM to enable them to learn influencing work and knowledge foundations.

**Figure 4:**
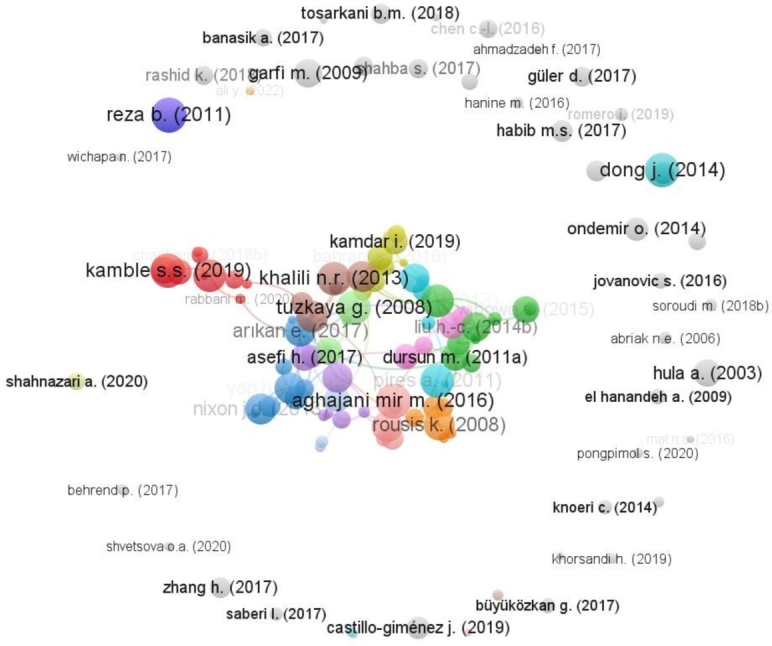
Document-wise Citation analysis.

Fig. 4 shows citations of reported work in the domain. Each work is referred by author name and year. We have taken the five minimum required citations, i.e., the articles must have received at least 5 citations. 123 of the total keywords are within the acceptable range. It is observed that less than twenty papers have more than 100 citations. The most cited work is of Reza B. (2011), which has been cited 132 times. The second most cited work is Aghajani Mir M. (2016). Both are empirical papers applying MCDM-based waste management. Reza B. in 2011 utilized the analytical hierarchy process (AHP), an MCDM technique, to create a sustainability index (SI) by aggregating the impacts of proposed (sub)criteria through a five-level hierarchical structure. Waste management was included as one of the main criteria. In contrast, Aghajani Mir M. in 2016 proposed improved versions of the Technique for Order of Preference by Similarity to Ideal Solution (TOPSIS) and Viekriterijumsko Kompromisno Rangiranje (VIKOR) in a multi-criteria decision analysis to develop an optimized model for municipal solid waste management. In addition, the articles with more than 100 citations include Tuzkaya G. (2008), Kamble S.S. (2019), Dong J. (2014), Pires A. (2011), Khalili N.R. (2013), Liu H.-C. (2013), Chauhan A. (2016), Coban A. (2018), Vučijak B. (2016), Rousis K. (2008), Vego G. (2008), Yap H.Y. (2015), Nixon J.D. (2013). These studies also suggested other MCDM techniques such as MULTIMOORA, TOPSIS, ELECTRE III, SEMS, Preference Ranking Organization Method for Enrichment Evaluations (PROMETHEE) I, and PROMETHEE II. Some of these studies employed a hybrid method of interpretive structural modeling, such as a fuzzy analytic hierarchy process and fuzzy technique for order preference and similarity to the ideal solution. Whereas Nixon's work has just received 100 citations only. Arıkan E. (2017), Karmperis A.C. (2013), and Liu H.-C. (2014) have been cited more than 90 times but less than 100. It is worth mentioning that all 14 articles with more than 100 citations have a publication duration of more than 5 years. There are 18 articles with citation ranges of 30-40, and 10 have citations between 20-30. There are 23 articles with a citation range of 10-20. In addition to this, there are 23 articles with less than 10 citations.

**Figure 5:**
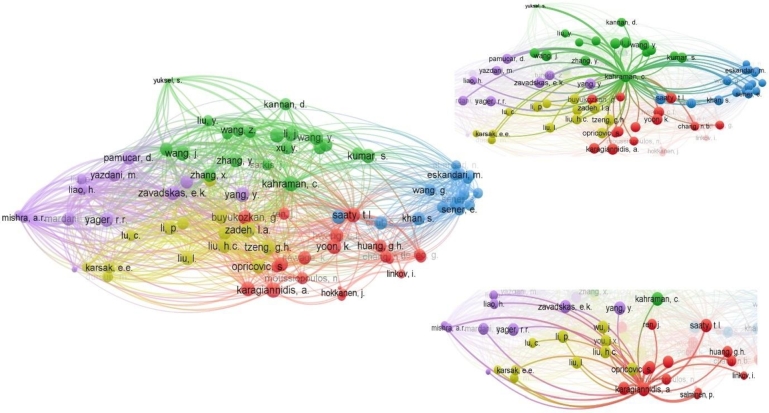
Represents co-citation analysis of cited authors, and most co-cited authors, and their collaboration network, i.e., mutually cited documents.

When we see the co-citation network of authors it is observed that 310 authors are co-cited at least 10 times and 82 of them are co-cited at least 20 times. It can be envisaged that their studies are very similar and coherent in terms of MCDM applications. Kahraman, C. and Karagiannidis, A. are the most co-cited authors with 80 different authors each. Kahraman, C. and Pamucar, D. are the two frequently co-cited authors cited 174 times together followed by Mishra, A.R. and Mardani, A. co-cited 161 times together. Mardani, A. and Rani, P. are also co-cited together 128 times making them the third most frequently co-cited authors.

**Figure 6:**
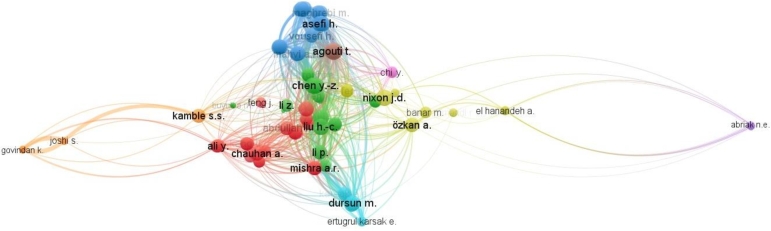
Analysis of bibliographic-coupling for authors.

Unlike co-citation, bibliographic coupling indicates documents citing the same articles. This can help in identifying related research fields and tracing the development of a research area over time. It helps us identify groups of highly-coupled publications and to measure the similarity of the work conducted in a particular field. For example, as shown in Fig. 6, the bibliographic coupling of authors can be analyzed. Nixon, J.D. leads the count with 65 bibliographic coupled documents i.e., there are 65 different documents that have very similar citations in their text as that of Nixon, J.D. The second most bibliographic coupled author is Liu, H.C. having 64 documents with similar citations. Fan, X.J., Chen, Y.Z. and You, J.X. are next in line with 63 different bibliographic coupled documents each. This implies the research theme of both authors has many similarities including similar references in their articles.

### 4.4 Scientific collaborations

Co-authorship analysis looks at how researchers connect with one another and analyzes the patterns of collaboration between authors. It provides insights into the collaboration networks among researchers and institutions, as well as the productivity and impact of these collaborations. It identifies the most productive collaborations, the most productive institutions, and the distribution of collaboration within and across disciplines. This information can be used to support the development of research strategies and to evaluate the impact of funding and policy decisions.

#### 4.4.1 Author analysis

To analyze authors' scientific collaboration, we have created an author co-occurrence diagram. Out of 677 authors, only 79 authors qualified the criteria of a minimum of two (2) published documents. To assess in-depth the strong collaboration, number of articles is desired to be more, however, for a better representation of the diagram to illustrate the collaboration network more conveniently, we have considered all the authors above minimum criteria. Seventy-nine (79) prolific authors and 36 clusters were classified by VOSViewer.

**Figure 7:**
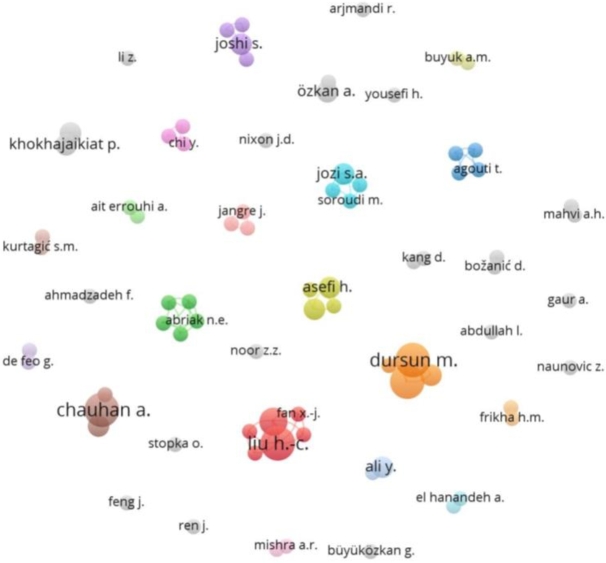
Author collaboration network with authors having a single publication is included.

The various clusters show the level of author collaboration, or how closely they are working together. (Fig. 7). The total link strength of this network is found to be 144. The total link strength indicates the total connection of an author with other authors in the network. Dursun, M and Karadayi, M.A. are the closest partners with highest link strength of 5 followed by You, J.X. and Liu, H.C., the second most collaborative partners with a link strength of 4. Ertugrul K.E. with Dursun, M and Karadayi, M.A and Lim, S with Asifi, H. ranked third with a link strength of 3 each. In terms of collaborating networks, red cluster represents the largest collaborating networks involving 6 authors, among which Liu H.C. and You, J.X. are the most collaborative ones, collaborating with 5 distinct authors, followed by the green cluster, having Gregoire, P, Abirak, N.E., Dubois, V, Junqua, G. and Damidot, D, with 4 different collaborating authors each. Beside these very few collaborating authors are linked with each other. Furthermore, there are 13 authors who have published two papers but have no collaborations. For example, H Yousefi has published articles as a single author [57].

#### 4.4.2 Institute analysis

**Figure 8:**
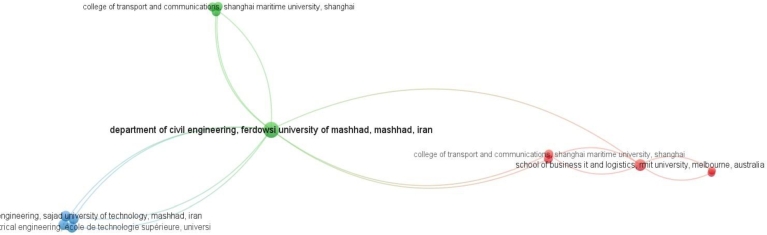
Collaboration network of institutions. Department of civil engineering, Ferdowsi University of Mashhad, Mashhad, Iran is the most collaborative, with 10 different collaborators.

We have depicted the current collaboration in Fig. 8 in order to analyze the scientific collaboration between various institutions and centers. Out of 479 institutes, 13 institutions with a total link strength of 25 comprise the greatest collaborating network. These institutions are arranged in 3 clusters. The Ferdowsi University of Mashhad's Department of Civil Engineering has the most institutional collaborations, with 10 different collaborators; it is followed by RMIT University's School of Business IT and Logistics, with 5 partner organizations. Sajad University of Technology (Mashhad), University of West London (UK), Tecnológico de Monterrey (Mexico), and École de technologie supérieure (Canada), each with four collaborations, make up the third largest collaborating network. With only two partnerships each, Central Queensland University in Australia and Renmin University in China are the least active collaborators. The remaining five institutions each have three collaborations. It is evident from the scenario that there is very little collaboration taking place when it comes to the work related to MCDM techniques-based waste management. Initiatives to forge closer ties could aid this area in developing further in the future because of the relatively low level of collaboration among these institutes. Increased global collaboration would improve information sharing and advance research in this field. More collaboration would not only improve their chances of coming to insightful conclusions about the topic, but would also help the field move forward in the future.

#### 4.4.3 Country analysis

**Figure 9:**
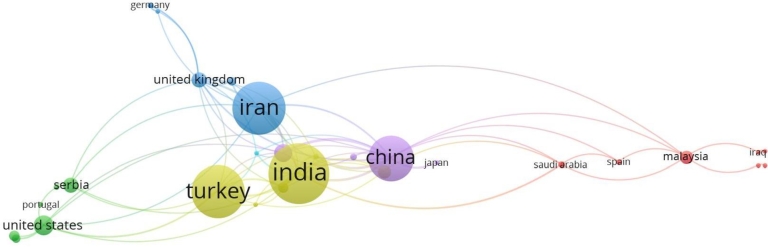
Analysis of co-authorship amongst countries.

To analyze the scientific collaboration among different countries, we have shown the existing collaboration via Fig. 9. Out of 56 countries, only 36 qualified the criteria of a minimum of two (2) published documents. Thirty (30) countries organized in 6 clusters having a total link strength of 102, were classified by VOSViewer.

As far as most active collaborators are concerned, China beats all, as they have collaborated with 15 different collaborators (countries), maximum for any country reported. China is followed by India and UK with 13 collaborating countries each. Next in line are Iran and Turkey, both having 10 collaborations. The most frequent collaborating partners are China and Australia with a link strength of 5 followed by China and India with a link strength of 4. Afterward, China with Turkey and Denmark, and India with UK are the next most collaborating countries with a link strength of 3 each.

### 4.5 Emerging keywords/research topics currently in consideration and future prospective

In this section, the keywords are analyzed which have been used in the concerned area of waste management and MCDM. Keyword co-occurrence analysis helps examine the relationships between keywords or terms within publications. The analysis is based on counting the frequency of co-occurrence of keywords in a publication's title, abstract, and keyword list. The more frequently two keywords occur together, the more closely related they will be in a research topic. As a result, it has become common practice to map the knowledge structure of a research topic using keyword co-occurrence to show that researchers' interests go beyond the subject at hand.

We have considered authors' keywords, index keywords and total keywords. The authors provide authors' keywords in the article, usually at the end of an abstract, index keywords are classified by Scopus [55], [56], and total keywords are a combination of these two categories as well as other keywords that may have been retrieved from various databases. To assess further the frequently occurring keywords, co-occurring keywords and total keywords as classified by the Scopus database, a different illustrative diagram which is created using VOSviewer has been presented.

The minimum number of times a term appears in this graph is 5. 164 of the 1917 keywords satisfy the criteria. There are 164 keywords in all that have been chosen. The term “decision-making” appears 160 times in this analysis. On the second and third, with 135 and 72 occurrences, waste management and multi-criteria decision-making, respectively. It is worth observing that the term “sustainability” appears 30 times on its own, and its related term “sustainable development” is found 37 times. In addition, when similar keywords such as “sustainable waste management,” “sustainability assessment,” “sustainability criteria,” “environmental sustainability,” “sustainable construction,” and “sustainable development goal” are taken into account, the count surpasses 72 instances. Alongside the appearance of “circular economy” and “environmental protection,” these keywords demonstrate a clear emphasis on sustainable practices.

The primary keyword is MCDM since it is linked to the bigger node. The second and third important keywords are waste management and TOPSIS. The top 5 keywords are decision making, waste management, MCDM, multicriteria analysis, and waste disposal, all of which are critical components of effective waste management systems. The use of Geographic Information Systems (GIS) (14 occurrences) and decision support systems (DSS) (20 occurrences) is also evident, which highlights the role of technology in waste management. Additionally, there is an emphasis on waste treatment methods such as “anaerobic digestion”, “composting”, and “incineration”. Overall, the keywords reflect a multidisciplinary approach to waste management that considers economic, social, and environmental factors.

It is envisaged that MCDM techniques are widely used in waste management research. These techniques help in solving complex decision-making problems by evaluating alternatives based on multiple criteria, and they help in identifying the best alternative by considering the trade-offs among different criteria. Some of the most common MCDM techniques mentioned in the list include “Analytic Hierarchy Process (AHP)”, “Multi-Attribute Utility Theory (MAUT)”, “Technique for Order Preference by Similarity to Ideal Solution (TOPSIS)”, “VlseKriterijumska Optimizacija I Kompromisno Resenje (VIKOR)” and “Analytic Network Process (ANP)”.

**Figure 10:**
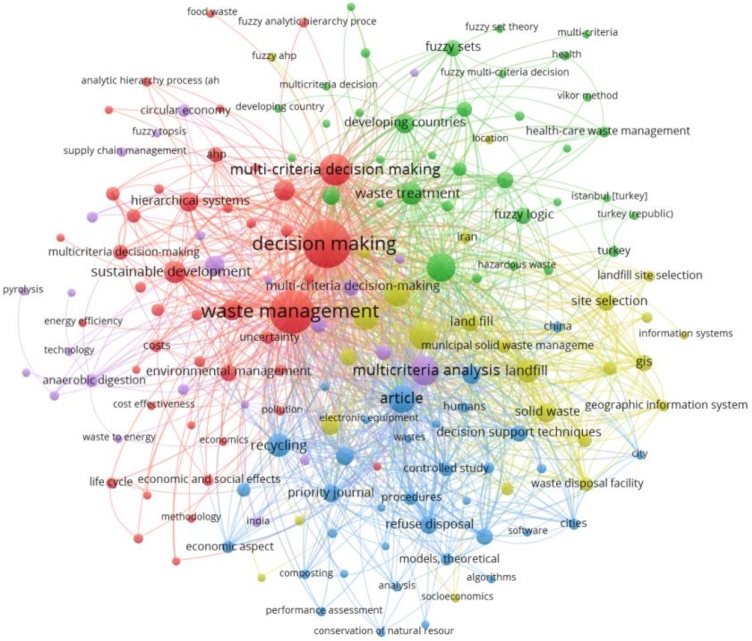
Analysis of co-occurrence of total keywords.

**Figure 11:**
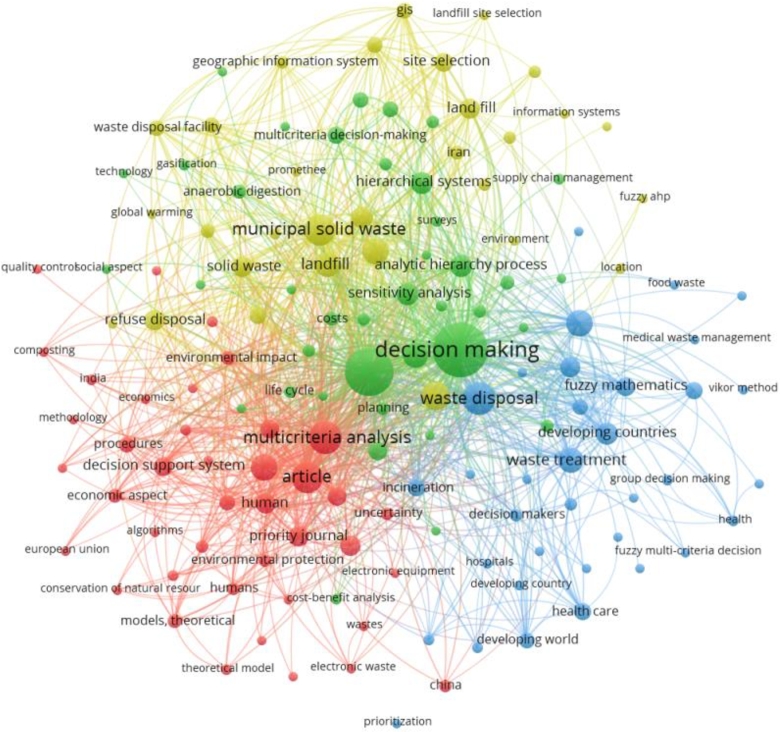
Analysis of co-occurrence of index keywords.

**Figure 12:**
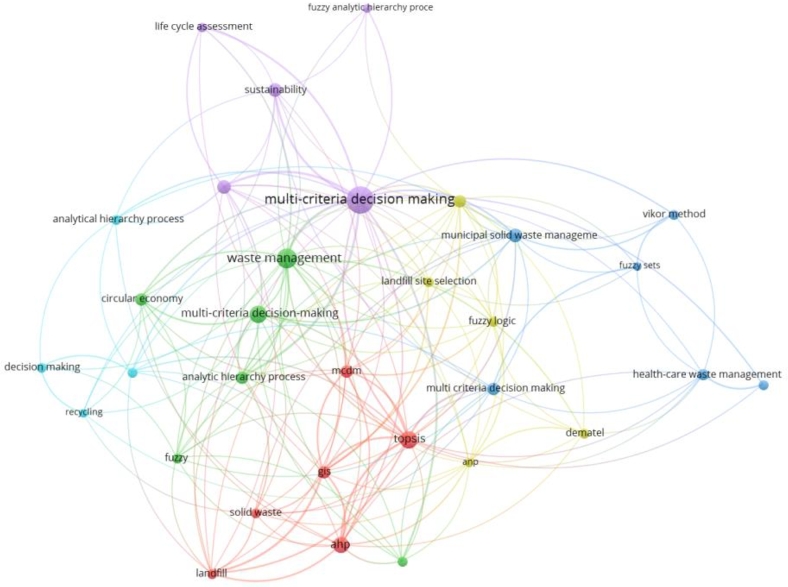
Analysis of co-occurrence of author's keywords.

From the above three diagrams, i.e., Fig. 10, Fig. 11 and Fig. 12, it can also be inferred that the keywords used by authors have less link strength and connectivity, and total number of keywords that satisfy the criteria are relatively less too. It implies that authors have not stated many frequent terms as their main keywords which have been classified by Scopus or other databases. Additionally, these terms can be explored and an investigation into how the occurrence of those words and their exploration in the concerned area impacts the growth of the research would be interesting.

**Figure 13:**
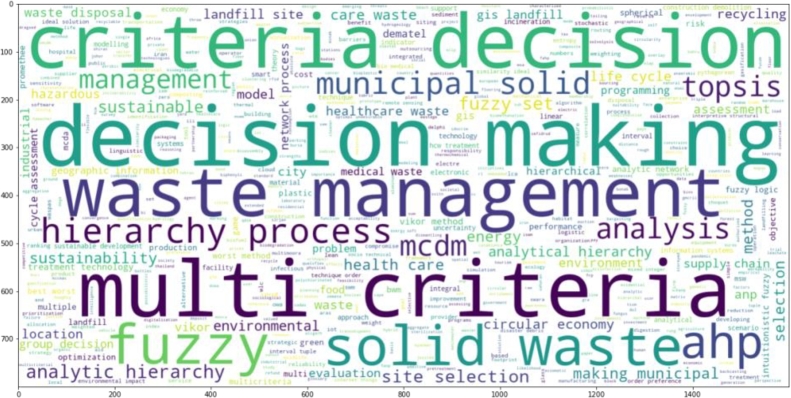
The emerging area identification via exploring subject-wise publication.

The primary search query terms that we used in the exploration of the manuscript to be considered in this review include waste management and MCDM; therefore, identifying other unexplored keywords that have been exploited in recent days shall give in-depth insights and would be instrumental to unearth the future research tone. To strengthen the keywords used and possible areas where works are being carried out, a word cloud is shown in Fig. 13 It is observed that similar to the keyword occurrence analysis, decision-making methods like TOPSIS, ANP and AHP are found to be evident from the word cloud. In addition to the methods, the research area in which works are carried out is also visible, for instance, municipal solid can be seen in big bold in the diagram, suggesting its involvement in the recent works in the domain. Similarly, solid waste can be seen in bold, indicating its greater importance in the concerned research.

**Figure 14:**
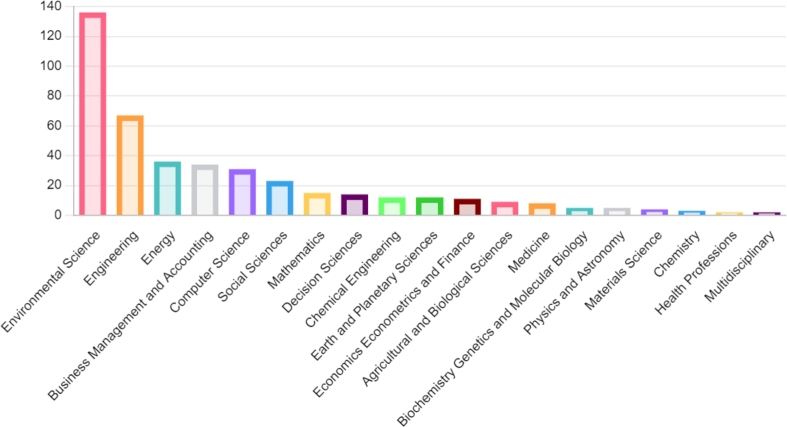
Distribution of subjects on the basis of research area related to concerned topic.

These keywords belong to different disciplines viz. environmental science, sustainability, engineering, management, medicine, and related field, etc. A bar graph in Fig. 14 displays the subject-wise examination of publications on MCDM and waste management. There have been a maximum of 136 publications on the subject of “environmental science.” Engineering has 67 publications, which is the second-most in number. Energy comes in third with a total of 36 articles, followed by “Business, Management and Accounting” and “Computer Science”. The least involved areas are “health profession” and “multidisciplinary,” with only 2 articles in these categories. This implies that waste management can play a significant role in environmental science growth, and from an ecological perspective, waste management is being investigated well. Moreover, engineering and energy are other areas where research opportunities can be explored. The wide distribution of topics among various subject areas could be due to the integrative approach of machine learning in waste management which in turn falls into other subject categories like environmental science, sustainability etc. Furthermore, as the number of articles in the health profession for the concerned area is least reported, it would be challenging for the professionals in the field to explore how waste management can contribute to these sections of society.

**Figure 15:**
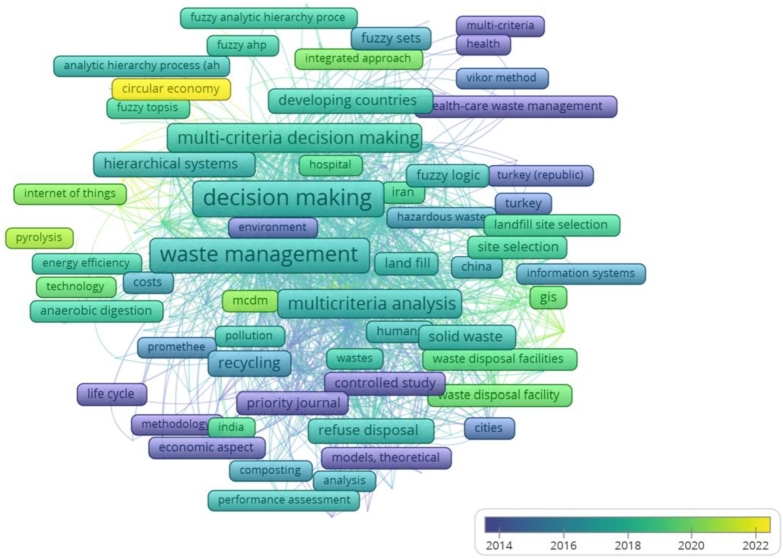
Evolution of keywords co-occurrence over a time-span, Blue color showing keywords occurrence prior to 2014, however, topics in green color are primarily evolved and had an impact after 2017-2018, and more recently occurred keywords are in yellow.

However, all the above figures support in identifying the co-occurring keywords and frequently used keywords to explore the future prospect of the research in the concerned field; a timeline of the occurring keywords is important. It enables the current research trends and helps identify the prime area in which research can be led. To his end, we have shown an overlay diagram in Fig. 15. The evolution of keywords co-occurrence over a time span has been presented. Blue color shows keywords occurrence prior to 2014; however, topics in green color primarily evolved during 2017-2018 and had an impact in this period. The most recent occurring keywords are in yellow. For instance, terms like “MCDM” or “multi-criteria or decision making” have been extensively used throughout the literature; however, “circular economy”, “pyrolysis”, “internet of things”, and “waste disposal facilities” are the keywords that recently have been explored and attracted the researchers. This can be inferred that these areas can be potentially explored in the near future. For example, the circular economy deals with waste management and climate change-related issues.

## 5 Conclusion

The research trends in the domain of waste management using MCDM techniques are evaluated in the present work for the period 1992-2022. Over the last decade, there has been a lot of research on this field of study, and the number of publications is growing exponentially. Our work presents an analysis of 216 works from 160 authors across 158 institutes in 56 countries. The analysis revealed useful information such as:

• Chauhan, A., Dursun, M., Karadayi, M.A. and Liu, H.C. published the highest number of works. Out of them, Liu, H.C. is the most productive in terms of citations.
• The primary journals that publish research on waste management using MCDM techniques are “Journal of cleaner production” and “Waste Management”.
• The countries that dominate the production of articles are India, Iran, Turkey, and China. Asian continent produces the majority of publications.
• Majority of the leading institutes are hailed from Turkey and Iran. Galatasaray Üniversitesi, Turkey, and Islamic Azad University, Iran top this list.
• Chinese institutes and researchers produce the most productive research in terms of number of citations and number of citations per article.
• AHP and Fuzzy-AHP, followed by TOPSIS, turned out to be the most commonly used MCDM approaches.

To conclude, it is important to mention some of the limitations of the current study. The search was restricted to Scopus-listed papers. Despite being one of the biggest databases in the world, Scopus obviously does not include all publications in this field of study. There were other worldwide databases that could have been utilized, such as Web of Science or PubMed. However, the most commonly used resource for analyzing scientific articles is Scopus. Moreover, quantitative techniques are used in the bibliometric analysis. Therefore, it is impossible to interpret the quality or content of articles. The fact that analysis can only be done for the available classifications included in Scopus was another constraint. As a result, some crucial aspects are left out, such as the distinction between empirical and theoretical works. In light of outlined limitations, a more thorough content analysis can be carried out for future studies.

In addition to this, the present study also reveals that MCDM approaches to circular economy could be a potential research topic that researchers shall work on, as it is posited in section 4.5. Since the overall goal of the circular economy is to utilize the resources efficiently and comprehensively. To achieve this goal, funding agencies and policymakers shall invest heavily in the development and implementation of new Industry 4.0 approaches to boost waste management. Moreover, circular supply chains and sustainability could be other promising and pertinent areas that might get more attention in the near future.

## CRediT authorship contribution statement

**Shahab Saquib Sohail:** Conceptualization, Methodology, Project administration, Supervision, Writing – original draft, Writing – review & editing. **Ziya Javed:** Resources, Writing – original draft, Writing – review & editing. **Mohammad Nadeem:** Conceptualization, Methodology, Writing – original draft, Writing – review & editing. **Faisal Anwer:** Resources, Writing – original draft, Writing – review & editing. **Faiza Farhat:** Resources, Writing – original draft, Writing – review & editing. **Amir Hussain:** Resources, Supervision. **Yassine Himeur:** Validation, Writing – original draft, Writing – review & editing. **Dag Øivind Madsen:** Project administration, Writing – review & editing, Validation.

## Declaration of Competing Interest

The authors declare that they have no known competing financial interests or personal relationships that could have appeared to influence the work reported in this paper.

## Data Availability

Data will be made available on request.

## References

[1] Mandpe Ashootosh, Paliya Sonam, Gedam Vidyadhar V., Patel Shubham, Tyagi Lakshay, Kumar Sunil (2023). Circular economy approach for sustainable solid waste management: a developing economy perspective. Waste Manag. Res..

[2] Sayed Aya, Himeur Yassine, Bensaali Faycal, Amira Abbes (2022). Emerging Real-World Applications of Internet of Things.

[3] Ragasri S., Sabumon P.C. (2023). A critical review on slaughterhouse waste management and framing sustainable practices in managing slaughterhouse waste in India. J. Environ. Manag..

[4] Rajaeifar Mohammad Ali, Ghanavati Hossein, Dashti Behrouz B., Heijungs Reinout, Aghbashlo Mortaza, Tabatabaei Meisam (2017). Electricity generation and ghg emission reduction potentials through different municipal solid waste management technologies: a comparative review. Renew. Sustain. Energy Rev..

[5] Seadon Jeffrey K. (2010). Sustainable waste management systems. J. Clean. Prod..

[6] Himeur Yassine, Rimal Bhagawat, Tiwary Abhishek, Amira Abbes (2022). Using artificial intelligence and data fusion for environmental monitoring: a review and future perspectives. Inf. Fusion.

[7] Coban Asli, Ertis Irem Firtina, Cavdaroglu Nur Ayvaz (2018). Municipal solid waste management via multi-criteria decision making methods: a case study in Istanbul, Turkey. J. Clean. Prod..

[8] Hoornweg Daniel, Bhada-Tata Perinaz (2012).

[9] Ebadi Torkayesh Ali, Ali Rajaeifar Mohammad, Rostom Madona, Malmir Behnam, Yazdani Morteza, Suh Sangwon, Heidrich Oliver (2022). Integrating life cycle assessment and multi criteria decision making for sustainable waste management: key issues and recommendations for future studies. Renew. Sustain. Energy Rev..

[10] Bhakta Sharma Kumar Raja Vanapalli Hari, Shankar Cheela V.R., Prakash Ranjan Ved, Kumar Jaglan Amit, Dubey Brajesh, Goel Sudha, Bhattacharya Jayanta (2020). Challenges, opportunities, and innovations for effective solid waste management during and post Covid-19 pandemic. Resour. Conserv. Recycl..

[11] Varlamis Iraklis, Sardianos Christos, Chronis Christos, Dimitrakopoulos George, Himeur Yassine, Alsalemi Abdullah, Bensaali Faycal, Amira Abbes (2022). Smart fusion of sensor data and human feedback for personalized energy-saving recommendations. Appl. Energy.

[12] Himeur Yassine, Al-Maadeed Somaya, Almaadeed Noor, Abualsaud Khalid, Mohamed Amr, Khattab Tamer, Elharrouss Omar (2022). Deep visual social distancing monitoring to combat Covid-19: a comprehensive survey. Sustain. Cities Soc..

[13] Kumar Patel Anil, Singhania Reeta Rani, Albarico Frank Paolo Jay B., Pandey Ashok, Chen Chiu-Wen, Dong Cheng-Di (2022). Organic wastes bioremediation and its changing prospects. Sci. Total Environ..

[14] Sardianos Christos, Varlamis Iraklis, Chronis Christos, Dimitrakopoulos George, Alsalemi Abdullah, Himeur Yassine, Bensaali Faycal, Amira Abbes (2021). The emergence of explainability of intelligent systems: delivering explainable and personalized recommendations for energy efficiency. Int. J. Intell. Syst..

[15] Achillas Charisios, Moussiopoulos Nicolas, Karagiannidis Avraam, Banias Georgias, Perkoulidis George (2013). The use of multi-criteria decision analysis to tackle waste management problems: a literature review. Waste Manag. Res..

[16] Coelho Lineker M. Goulart, Lange Liséte C., Coelho Hosmanny M.G. (2017). Multi-criteria decision making to support waste management: a critical review of current practices and methods. Waste Manag. Res..

[17] Yazdani Morteza, Chatterjee Prasenjit, Zavadskas Edmundas Kazimieras, Zolfani Sarfaraz Hashemkhani (2017). Integrated qfd-mcdm framework for green supplier selection. J. Clean. Prod..

[18] Ishizaka Alessio, Siraj Sajid (2018). Are multi-criteria decision-making tools useful? An experimental comparative study of three methods. Eur. J. Oper. Res..

[19] Bhagtani Neha (2008).

[20] Hajkowicz Stefan, Higgins Andrew (2008). A comparison of multiple criteria analysis techniques for water resource management. Eur. J. Oper. Res..

[21] Ebadi Torkayesh Ali, Fathipoir Fariba, Saidi-Mehrabd Mohammad (2019). Entropy-based multi-criteria analysis of thermochemical conversions for energy recovery from municipal solid waste using fuzzy vikor and electre iii: case of Azerbaijan region, Iran. J. Energy Manag. Technol..

[22] Mardani Abbas, Jusoh Ahmad, Zavadskas Edmundas Kazimieras (2015). Fuzzy multiple criteria decision-making techniques and applications–two decades review from 1994 to 2014. Expert Syst. Appl..

[23] Ekundayo Temitope C., Igwaran Aboi, Oluwafemi Yinka D., Okoh Anthony I. (2021). Global bibliometric meta-analytic assessment of research trends on microbial chlorine resistance in drinking water/water treatment systems. J. Environ. Manag..

[24] Santos Murilo R., Dias Luis C., Cunha Maria C., Marques João R. (2022). Multicriteria decision analysis addressing marine and terrestrial plastic waste management: a review. Front. Mar. Sci..

[25] Cheng Steven, Chan Christine W., Huang Guo H. (2002). Using multiple criteria decision analysis for supporting decisions of solid waste management. J. Environ. Sci. Health, Part A.

[26] Abu R., Aziz M.A.A., Sapuan N., Abdullah T.A.T., Hassan C.H.C., Noor Z.Z. (2021).

[27] Marinello Samuele, Gamberini Rita (2021). Multi-criteria decision making approaches applied to waste electrical and electronic equipment (weee): a comprehensive literature review. Toxics.

[28] Soltani Atousa, Hewage Kasun, Reza Bahareh, Sadiq Rehan (2015). Multiple stakeholders in multi-criteria decision-making in the context of municipal solid waste management: a review. Waste Manag..

[29] Donthu Naveen, Kumar Satish, Mukherjee Debmalya, Pandey Nitesh, Lim Weng Marc (2021). How to conduct a bibliometric analysis: an overview and guidelines. J. Bus. Res..

[30] Gauthier Élaine (1998).

[31] Farhat Faiza, Sohail Shahab Saquib, Siddiqui Farheen, Irshad Reyazur Rashid, Madsen Dag Øivind (2023). Curcumin in wound healing—a bibliometric analysis. Life.

[32] Van Eck Nees, Waltman Ludo (2010). Software survey: vosviewer, a computer program for bibliometric mapping. Scientometrics.

[33] Van Eck Nees Jan, Waltman Ludo (2011). Text mining and visualization using vosviewer. arxiv:1109.2058.

[34] Shah Syed Hamad Hassan, Lei Shen, Ali Muhammad, Doronin Dmitrii, Hussain Syed Talib (2020). Prosumption: bibliometric analysis using histcite and vosviewer. Kybernetes.

[35] Chandel Ajay, Kaur Tejbir (2022). Developing Relationships, Personalization, and Data Herald in Marketing 5.0.

[36] Soegoto Eddy Soeryanto, Neni Hayati Euis, Untsa Mega Raiswati, Ihsan Rifaldi Muhammad (2023). Economic growth and its influence on environment sustainability: a bibliometric analysis using vosviewer application. J. East.-Eur. Cent. Asian Res..

[37] Van Eck Nees Jan, Waltman Ludo (2017). Citation-based clustering of publications using citnetexplorer and vosviewer. Scientometrics.

[38] Sood Sandeep Kumar, Kumar Navin, Saini Munish (2021). Scientometric analysis of literature on distributed vehicular networks: vosviewer visualization techniques. Artif. Intell. Rev..

[39] Hou Yukun, Yu Zhonggen (2023). A bibliometric analysis of synchronous computer-mediated communication in language learning using vosviewer and citnetexplorer. Educ. Sci..

[40] Bartol Tomaz (2023). Smallholders and small-scale agriculture: mapping and visualization of knowledge domains and research trends. Cogent Soc. Sci..

[41] Catani Linda, Grassi Eleonora, Cocozza di Montanara Adele, Guidi Loretta, Sandulli Roberto, Manachini Barbara, Semprucci Federica (2022). Essential oils and their applications in agriculture and agricultural products: a literature analysis through vosviewer. Biocatal. Agric. Biotechnol..

[42] Luckyardi S., Soeryanto Soegoto E., Jumansyah R., Puspa Dewi N., Untsa Mega R. (2022). A bibliometric analysis of climate smart agriculture research using vosviewer. Moroc. J. Chem..

[43] Contreras Francoise, Abid Ghulam (2022). Social sustainability studies in the 21st century: a bibliometric mapping analysis using vosviewer software. Pak. J. Commer. Soc. Sci..

[44] Abdullah Khairul Hafezad (2022). Mapping of literature on safety knowledge research using scientopy and vosviewer. J. Metr. Stud. Soc. Sci..

[45] Jia Chenjin, Mustafa Hasrina (2022). A bibliometric analysis and review of nudge research using vosviewer. Behav. Sci..

[46] Feng Yang, Gu Xinyuan, Ye Jianqing, Jia Xiaolin, Zhang Hongchen, Wang Sirong, Yang Jianfeng (2022).

[47] El Wahab Mostafa H. Abd (2022). Covid-19 and curcumin: using vosviewer software to explore scientific landscape, a bibliometric analysis. medRxiv.

[48] Yeung Andy Wai Kan, Tosevska Anela, Klager Elisabeth, Eibensteiner Fabian, Tsagkaris Christos, Parvanov Emil D., Nawaz Faisal A., Völkl-Kernstock Sabine, Schaden Eva, Kletecka-Pulker Maria (2022). Medical and health-related misinformation on social media: bibliometric study of the scientific literature. J. Med. Internet Res..

[49] Li Desheng, Zuo Minfang, Hu Xinming (2022). Global trends in research of treatment on bladder cancer with Chinese medicine monomer from 2000 to 2021: a bibliometric analysis. J. Oncol..

[50] Van Eck Nees Jan, Waltman Ludo, Dekker Rommert, Van Den Berg Jan (2010). A comparison of two techniques for bibliometric mapping: multidimensional scaling and vos. J. Am. Soc. Inf. Sci. Technol..

[51] Waltman Ludo, Van Eck Nees Jan, Noyons Ed C.M. (2010). A unified approach to mapping and clustering of bibliometric networks. J. Informetr..

[52] Rizzi Francesco, van Eck Nees Jan, Frey Marco (2014). The production of scientific knowledge on renewable energies: worldwide trends, dynamics and challenges and implications for management. Renew. Energy.

[53] Chakraborty Sayanta, Saha Apu Kumar (2022). A framework of lr fuzzy ahp and fuzzy waspas for health care waste recycling technology. Appl. Soft Comput..

[54] Khalil George M., Crawford Carol A. Gotway (2015). A bibliometric analysis of us-based research on the behavioral risk factor surveillance system. Am. J. Prev. Med..

[55] Merigó José M., Gil-Lafuente Anna M., Yager Ronald R. (2015). An overview of fuzzy research with bibliometric indicators. Appl. Soft Comput..

[56] Purwanto Agus, Fahmi Khaerul, Cahyono Yoyok (2023). Online workshop of article writing for scopus indexed journals. J. Community Serv. Engagement.

[57] Yousefi Hossein (2009). Canola straw as a bio-waste resource for medium density fiberboard (mdf) manufacture. Waste Manag..

